# Birth Cohorts in Highly Contaminated Sites: A Tool for Monitoring the Relationships Between Environmental Pollutants and Children's Health

**DOI:** 10.3389/fpubh.2020.00125

**Published:** 2020-04-28

**Authors:** Gaspare Drago, Silvia Ruggieri, Fabrizio Bianchi, Silvestre Sampino, Fabio Cibella

**Affiliations:** ^1^National Research Council of Italy, Institute for Biomedical Research and Innovation, Palermo, Italy; ^2^National Research Council of Italy, Institute of Clinical Physiology, Pisa, Italy; ^3^Department of Experimental Embryology, Institute of Genetics and Animal Breeding of the Polish Academy of Sciences, Jastrzebiec, Poland

**Keywords:** birth cohorts, highly contaminated sites, fetal exposome, DOHaD postulate, placenta, child growth

## Abstract

Industrial areas are characterized by the dispersion of environmental stressors that could possibly have long-term detrimental effects on both human health and the environment. Environmental contamination has been indicated to be one of the major risks for reproductive health. In this context, the effects of environmental pollution on pregnant women living in heavily polluted areas is of special interest. In fact, fetal development is a crucial phase due to the dynamic interaction between the maternal/external environments and the developing organs and tissues. Moreover, following Barker's postulate of the intrauterine origin of health and disease, the events occurring in this time window could affect future health. Birth cohorts provide the most suitable design for assessing the association between early-life and possible long-term health outcomes in highly contaminated sites. By providing an assessment of the early life environment throughout the collection of biological samples, birth cohorts offer the opportunity to study in-depth several possible confounders and outcomes by means of questionnaires and follow-ups based on clinical evaluations and bio-specimen samplings. The exposome comprises the totality of exposures from conception onwards; the birth cohort approach allows the integration of the exposures as a whole, including those related to socioeconomic status, with “omics” data from biological samples collected at birth and throughout life. In the characterization of the “fetal exposome,” the placenta represents a highly informative and scarcely considered organ. For this purpose, the “Neonatal Environment and Health Outcomes” (NEHO) birth cohort has been established by enrolling pregnant women residing in contaminated sites and in surrounding areas.

## Introduction

### Health Effects of Environmental Exposures in Highly Contaminated Sites

Contaminated sites can be defined as “areas hosting or having hosted human activities which have produced or might produce environmental contamination of soil, surface or groundwater, air, food-chain, resulting or being able to result in human health impacts” (1). Contaminants such as heavy metals (HMs) and persistent organic pollutants (POPs) may transfer from one environmental matrix to another and, depending on their chemical-physical properties, are able to infiltrate the human body through different exposure pathways and routes (2). Living near environmental hazards contributes to a poorer general health status (3). Moreover, contaminated sites are often located in socially deprived neighborhoods; this makes exposure patterns more complex and results in interactions with other health determinants (4). Environmental pollution is one of the largest causes of death and disability in the world. In 2015, about 16% of premature deaths worldwide were caused by exposure to chemicals released into the environment (5). The WHO highlighted that 26% of deaths among children under five are due to modifiable environmental factors, and therefore can be prevented (6). Moreover, many childhood morbidities and disabilities are attributable to environmental causes and to gene-environment interaction starting from the fetal development period (7).

Environmental contamination is one of the major risk factors for reproductive health (8). Indeed, causal relationships between parental or prenatal environmental exposures and several adverse pregnancy and childhood outcomes have been clearly documented (9–13). Moreover, toxicants were associated with intrauterine growth restriction (14–16), inadequate birth weight (17), and premature births (18). These birth outcomes are of special interest for their double significance: they represent both an adverse outcome *per se* and could be considered as risk factors for future childhood pathologies.

Many chemicals released into the environment due to industrial processes are able to disrupt the programming of endocrine signaling. Thus, gametes, pregnant women, and developing fetuses are particularly vulnerable to the harmful impact of these environmental toxicants (19–21). For example, cadmium (Cd) has been identified as an endocrine disruptor and is released by industrial plants, negatively influencing both male and female reproductive health, acting at the level of the hypothalamic-pituitary-gonadal intercommunication axis (22). Lastly, human studies have shown an association between Cd exposure during pregnancy and low birth weight (23).

There is growing evidence supporting the hypothesis that prenatal exposure to toxicants is associated with long-term effects on children's neurological development (24, 25), respiratory and cardiovascular systems (26), and metabolic signaling (27). For instance, children born to women exposed to organochlorine pesticides have a higher risk of developing neurodevelopmental, neurodegenerative, and neurobehavioral disorders (28, 29). Prenatal exposure to methylmercury has also been associated with the development of autism spectrum disorders (30). Similarly, a significant increase in the incidence of “bronchitis” was reported in Taiwanese children born to women exposed to polychlorinated biphenyls (PCBs) during pregnancy (31). Moreover, prenatal exposure to perfluoroalkyl substances and postnatal exposure to copper, ethylparaben, and household crowding were associated with poorer lung function in 6- and 12-year-old children (32). *In-utero* exposure to hexachlorobenzene (HCB) and dichlorodiphenyldichloroethylene (DDE) was associated with childhood obesity and higher blood pressure levels at 4 years (33). In this study, an obesogenic effect of DDE and HCB was hypothesized through sex steroid dysregulation. Moreover, in a French birth cohort, an association was found between high maternal Cd and lead (Pb) blood levels and increased risk of gestational diabetes (34). Fetal exposure to maternal gestational diabetes was further associated with an altered glucose-induced hypothalamic activity in children and, as a consequence, with increased risk of obesity later in life (35).

Scientific data on the long-term effects of developmental exposures provide new insight into the importance of preventing the negative effects of environmental chemicals on the residents of highly polluted sites. There are different methodologies to assessing pollutant impacts on human health through studies with both ecological and etiological design. One example is the SENTIERI Project, which works toward multiple endpoints, including hospital discharges during infancy and congenital anomalies, in all the main contaminated sites in Italy, implementing an a-priori identification of health endpoints linked with pollution sources (36). This approach is of undeniable value for public health monitoring, even though any demonstration of pathophysiological links between environmental pollutants and health effects requires further research.

It is generally recognized that prospective pregnancy or birth cohort studies, incorporating exposure biomarkers during sensitive windows, are required to examine the potential health effects of developmental exposure to chemicals. Birth cohort studies provide the most suitable design for assessing the association of early-life adversities occurring at critical developmental windows with their possible long-lasting effects on postnatal health and well-being. Cohort populations living in highly contaminated sites have been studied mainly in occupational settings; in contrast, their use in the general population is not well-represented in scientific literature, though remarkable examples are available, especially in cases of accidental events, such as the Seveso disaster (37) or Minamata disease (38).

### Fetal Development and Environmental Epidemiology

Starting from Barker's postulate of the “intrauterine origins of health and disease susceptibility” (39–41), growing evidence has highlighted how environmental stressors can interfere with the early stages of fetal development leading to diseases later in life. Chemical compounds, social stress, and lifestyle can lead to the permanent alteration of fetal development, possibly resulting in increased susceptibility to adverse health outcomes over a person's lifetime (42, 43). Homeostatic processes during fetal life allow the organism to dynamically adapt to changes in the intrauterine environment in order to obtain an immediate survival chance and to have future adaptive advantages in adulthood (44). However, changes that turn out to be adaptive for one endpoint, such as surviving an acute stressful condition, may be maladaptive in other life stages, thus producing a higher risk of non-communicable disease occurrence (45).

In recent years, the role of epigenetic mechanisms (e.g., nucleotide and histone chemical modifications and small non-coding RNAs) has been recognized in regulating fetal development and its adaptation to changing environmental conditions through changing gene expression (45), while growing evidence has drawn attention to epigenetic alterations induced by environmental contaminants (46). Epigenetic alterations that affect the trajectories of fetal development may maintain their effects over generations (47). Industrial activities and power plants are known sources of many chemicals which can induce epigenetic effects (2, 48–52). DNA methylation is a potential mechanism by which environmental exposures may contribute to the etiology of complex diseases (53). Epigenetic changes have been observed in pregnant women, placentas, and cord blood after exposure to various environmental contaminants, such as phthalate and bisphenol A (54), but also to maternal smoking (55) and psychological stress (56). In a large-scale epigenome-wide meta-analysis, the authors found a significant association of PM_10_ and PM_2.5_ exposures during pregnancy with methylation differences in newborns' genes relevant to respiratory health, such as FAM13A and NOTCH4 (57).

**Figure 1:**
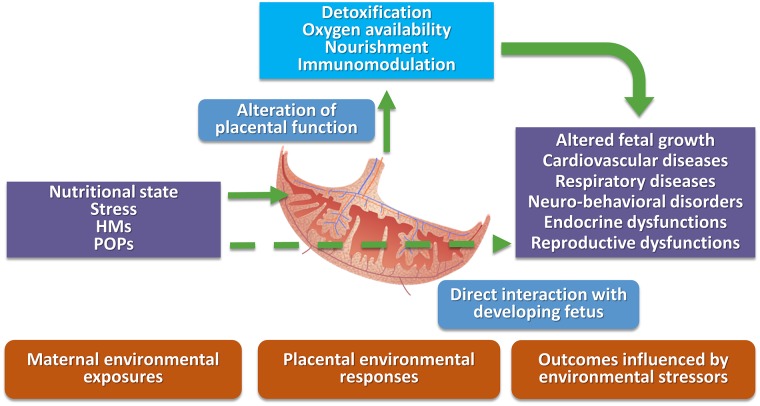
Schematic representation of direct and indirect interactions among environmental stressors, placental domain, and possible negative health outcomes. HMs, heavy metals; POPs, persistent organic pollutants.

Pregnant women living in highly contaminated sites can be exposed via multiple pathways, including food, inhalation, and dermal contact. The exposure of the developing fetus to environmental contaminants may lead to multi-organ alterations producing organ dysfunction and diseases. Toxicants influence fetal development in different ways. The influence can be direct, as in the case of arsenic (As), Pb, and mercury (Hg) as these substances can readily pass through the placenta into the fetal environment (58), or indirect, as in the case of Cd, by interfering with maternal and placental homeostatic functions leading in turn to altered signaling with the fetus (Figure 1). For other environmental toxicants, such as PCBs, their ability to pass through the placenta is related to congener specific chemical-physical properties, such as molecular weight and lipophilicity (59).

Many studies performed in highly contaminated areas have evaluated residential proximity to pollution sources or air pollutant exposures (11, 60–63). On the contrary, studies on the contribution of soil and water contamination to human exposure, as well as those related to the food chain and human biomonitoring, are less represented in scientific literature (64–66).

Recently, Heindel and colleagues published a comprehensive review of epidemiological studies evaluating associations between *in-utero* and early post-natal life exposure to environmental chemicals and adverse health outcomes (67). They examined 425 papers, showing that most of the publications were related to neurological/cognitive outcomes, followed by cancer and respiratory diseases. Only in recent years have researchers focused on metabolic outcomes (including obesity) and second generation reproductive health (67). Similarly, studies in highly contaminated sites have indicated a greater incidence and prevalence of a variety of health conditions, including cancer, respiratory diseases, diabetes, obesity, and negative reproductive health outcomes (68, 69). Moreover, in Heindel's review, more than 60 different chemical compounds were identified, most of which are known to be related to environmental contamination due to power plants and industrial/petrochemical emissions. The most frequently studied chemicals are PCBs, often associated with incineration and power generation processes (70). Regarding heavy metals, Hg, Pb, and As were the most represented in the review. In Table 1, a short list of studies on environmental pollutants present in highly polluted areas is reported along with the relevant health outcomes in pediatric age (71–98).

**Table 1:** Selected list of studies on environmental pollutants performed in pediatric age in highly polluted areas, along with the relevant health outcomes.

**Exposure**	**Type of study**	**Sample characteristics and compounds measurements**	**Outcomes**	**References**
As	Retrospective cohort study	*in utero* and childhood exposure to As. Standard mortality rates calculated for populations living in contaminated areas compared to those of the rest of Chile.	Exposure through drinking water during early childhood or *in utero* increases mortality rate in young adults due to both malignant and non-malignant lung disease.	Smith et al. (71)
	Case-control study	339 women having children with congenital heart defects (CHDs) and 333 women with normal live births in China. As levels were measured in maternal hair samples.	Maternal exposure to As had a significant association with the risk of CHDs in offspring.	Jin et al. (72)
	Case-control study	435 women having children affected by oro-facial clefts and 1,267 mothers of unaffected children. As levels were estimates by questionnaire (occupational, drinking water, and dietary As exposure) along with a subsample of subjects with measures of individual exposure levels to As.	Positive association was observed for maternal occupational As exposure and cleft palate.	Suhl et al. (73)
	Cross-sectional study	Concentration of As in cord blood samples collected in 892 births.	Prenatal exposure to As was associated with poor neurobehavioral performance of newborns, particularly among those born to older mothers.	Wang et al. (74)
	Meta-analysis	Including 18 reports from cross-sectional, case-control and cohort studies of As exposure.	Authors reported that 50% increase of As levels in child urine would be associated with a 0.4 decrease in the intelligence quotient of children aged 5–15.	Rodríguez-Barranco et al. (75)
Hg	Meta-analysis	Meta-analysis was conducted for two major exposure sources: thimerosal vaccines that contain ethylmercury (clinical exposure) and environmental sources, using relevant literature published before April 2014.	Moderate adverse effects were observed only between environmental inorganic or organic Hg exposures and autism spectrum and attention deficit hyperactivity disorders. No effect of vaccine-derived Hg was observed.	Yoshimasu et al. (76)
	Cohort study	The Mediterranean (Italy, Slovenia, Croatia, and Greece) cohort study included 1,308 mother-child pairs. Hg levels were measured in different maternal biological samples and cord blood.	Inverse relation between Hg levels and child developmental motor scores at 18 months. No evidence of detrimental effects of Hg was found for cognitive and language outcomes.	Barbone et al. (77)
	Cohort study	Including 458 mother/infant pairs. Blood Hg levels were measured in cord blood at early and late pregnancy and at 2 and 3 years of age.	Blood Hg levels at late pregnancy and early childhood were associated with more severe autistic behaviors.	Ryu et al. (78)
	Cohort study	Maternal Hg blood concentration at 17th gestational week analyzed in 2,239 women of a Norwegian cohort.	A small but significant adverse association between children above the 90th percentile dietary Hg exposure and childhood language skills.	Vejrup et al. (79)
Cd	Cohort study	300 mothers in China. Maternal blood Cd concentration.	A 10-fold increase in maternal Cd levels was associated with a 5.7-point decrease in social domain developmental quotient and a 4.3-point decrease in circulating brain-derived neurotrophic factor levels.	Wang et al. (80)
	Cohort study	575 mother-child pairs from the prospective “Rhea” cohort on Crete, Greece. Exposure was estimated by maternal urine Cd concentrations during pregnancy.	Elevated urinary Cd concentrations (≥0.8 μg/L) were inversely associated with children's general cognitive score.	Kippler et al. (81)
	Cohort study	515 mother-child pairs from the “Rhea” cohort on Heraklion, Greece. Urinary Cd concentrations measured during early pregnancy.	Elevated prenatal Cd levels were significantly associated with a slower weight trajectory and a slower height trajectory in girls and in children born to mothers who smoked during pregnancy.	Chatzi et al. (82)
	Cohort study	Cd exposure was assessed by urinary concentrations during early pregnancy (*n* = 1,299), 5 (*n* = 1,453), and 10 years of age (*n* = 1,498).	Childhood Cd exposure was associated with lower intelligence in boys, and there were indications of altered behavior in girls for both prenatal and childhood exposures.	Gustin et al. (83)
	Cohort study	185 participants from the ELEMENT birth cohorts in Mexico City with complete data on urinary Cd exposures, anthropometry and covariates.	Prenatal Cd exposure was negatively associated with measures of both abdominal and peripheral adiposities in girls, but not in boys.	Moynihan et al. (84)
Pb	Cohort study	4,285 pregnant women from the ALSPAC cohort. Pb levels were analyzed in blood samples from pregnant women and from 235 children at age of 30 months.	Prenatal Pb exposure was not significantly associated with child IQ at 4 or 8 years. However, some evidence suggests that boys are more susceptible than girls to prenatal exposure to Pb.	Taylor et al. (85)
	Cohort study	965 pregnant women. Information about dietary intake, and maternal and cord blood levels were collected for Pb exposure assessment.	Maternal late pregnancy Pb was marginally associated with deficits in mental development index of children at 6 months.	Shah-Kulkarni et al. (86)
	Cohort study	Pb was measured in 334 mid-pregnancy women, in 362 late-pregnancy women and in umbilical cord blood, in a cohort of full-term infants in rural northeastern China.	Auditory brainstem response (ABR) and grating visual acuity (VA) maturation appears delayed in infants with higher prenatal Pb exposure during late-pregnancy, even at relatively low levels.	Silver et al. (87)
	Cohort study	Pb and As were measured in 257 maternal toenail samples collected at 28 weeks gestation and/or in 285 samples 6 weeks postpartum.	*in utero* toxic metal exposures may be associated with early life increases in blood pressure in children, which could have consequences for long-term health.	Farzan et al. (88)
	Cohort study	Pb levels were measured between 20 to 24 weeks of pregnancy and in cord blood, in 402 children from the Polish Mother and Child Cohort (REPRO_PL).	Fetal exposure to very low Pb levels might affect early cognitive domain, with boys being more susceptible than girls.	Polanska et al. (89)
PCBs	Case-control study	Southern California births, including 545 children with autism spectrum disorders (ASD) and 181 with intellectual disability (ID), as well as 418 healthy children. Concentrations of 11 PCB congeners and 2 OCPs measured in second-trimester maternal serum samples.	Higher levels of organochlorine compounds during pregnancy are associated with ASD and ID.	Lyall et al. (90)
	Cohort study	PCB and DDE were measured in maternal serum and breast milk in 656 women.	Association of PCD and DDE levels with body-mass index of girl aged 5–7 years in relation to maternal body weight.	Tang-Péronard et al. (91)
	Cohort study	Concentration of 17 PCB congeners analyzed in umbilical blood cord samples, in a total of 40 healthy term pregnancies.	Association between PCB 118 concentration and fixation pattern examined by the upright and inverted biological motion (BM) test at 4-months after birth, as a measure of social functioning.	Doi et al. (92)
	Meta-analysis	Pooled data from seven European birth cohorts with biomarker concentrations of PCB-153 and DDE in 2,487 and 1,864 samples respectively.	Significant increase in growth associated with DDE, seemingly due to prenatal exposure, and significant decrease in growth was associated with postnatal PCB-153 exposure.	Iszatt et al. (93)
	Cohort study	Concentrations of PCBs and OH-PCBs were determined in cord blood samples of 97 mother-infant pairs.	Associations between PCB and OH-PCB levels and motor optimality score, including detailed aspects of the early motor development, measured at 3-month-old infants.	Berghuis et al. (94)
PAH	Cohort study	727 Dominican or African American women living in Northern Manhattan or the South Bronx were enrolled during pregnancy. Prenatal PAH exposure was measured from 48-h personal air monitoring, and children's PAH exposure at 5 to 6 years of age was measured from residential indoor monitoring.	Repeated high exposure to pyrene was positively associated with the development of asthma, ever wheeze, asthma medication use, and emergency department visits for asthma.	Jung et al. (95)
	Cohort study	727 Dominican or African American women living in Northern Manhattan or the South Bronx were enrolled during pregnancy. Prenatal PAH exposure was measured from 48-h personal air monitoring.	Higher prenatal PAH exposures were significantly associated with higher risk for obesity at 5 and 7 years of age.	Rundle et al. (96)
	Cohort study	353 women enrolled in Krakow, Poland with valid airborne PAH data. To assess exposure to PAHs, the women were personally monitored over a 48-h period during the second (*n* = 253) or third (*n* = 100) trimester of pregnancy.	Higher prenatal exposure to airborne PAHs was found associated with a statistically significant reduction in scores on a test of non-verbal child intelligence in 5-year-old children.	Edwards et al. (97)
	Cohort study	151 children from a birth cohort study conducted by the Columbia Center for Children's Environmental Health (CCCEH) residing in Krakow, Poland. Prenatal airborne PAH exposure was measured by personal air monitoring.	PAH measures from prenatal personal air monitoring was positively associated with adverse neurodevelopment in children.	Genkinger et al. (98)

*As, arsenic; Hg, mercury; Cd, cadmium; Pb, lead; PCB, polychlorinated biphenyls; PAH, polycyclic aromatic hydrocarbons*.

However, people living in heavily contaminated areas present a different exposure profile as compared to the general population, in terms of both level of exposure and number of contaminated environmental matrices. In this respect, multipollutant models are designed to overcome the difficulty of identifying effects of multiple pollutants in epidemiological studies which also try to effectively capture the health impact of pollution mixtures observed under real-life conditions. These models are of particular interest, though scarcely represented, especially in the context of highly contaminated sites. Moreover, the evaluation of real-life exposure represents a methodological challenge for the overall integration of exposure measures obtained from different matrices (e.g., ambient air, blood, tissues).

### The Fetal Exposome and the Placenta

Omics, including genome, epigenome, transcriptome, metabolome, and microbiome, have widened our ability to investigate complex biological processes. The possibility of considering multiple molecular pathways at once gives us the opportunity to have a more holistic and comprehensive understanding of an organism's development and functions. Along the same line, Christopher Paul Wild coined the term “exposome” in order to promote the use of an omics approach in the field of environmental epidemiology (99).

Consistent advances have been made in “measuring” the levels of environmental contaminants in biological tissues; however, a delineated exposome approach has not been applied in clinical settings. The exposome not only concerns toxic chemicals but also includes three domains: (1) a general external domain including the social and economic context and stress factors; (2) a specific external domain including environmental pollutants, diet, and drugs; and (3) a specific internal domain including biomarkers of exposure, effects, and susceptibility (100). Another key concept in defining the exposome is its dynamic nature (100): for example, changes in household, school, occupation, socioeconomic profile, social interactions and stress, course of medical treatment, exposure profile (even for a short period), and migration flows may all produce changes in the exposome during a lifetime and should be measured over time. Therefore, the full characterization of an individual's exposome requires a number of measures able to capture exposure during their lifespan. However, individual susceptibility changes with age, and specific time windows can be identified.

**Figure 2:**
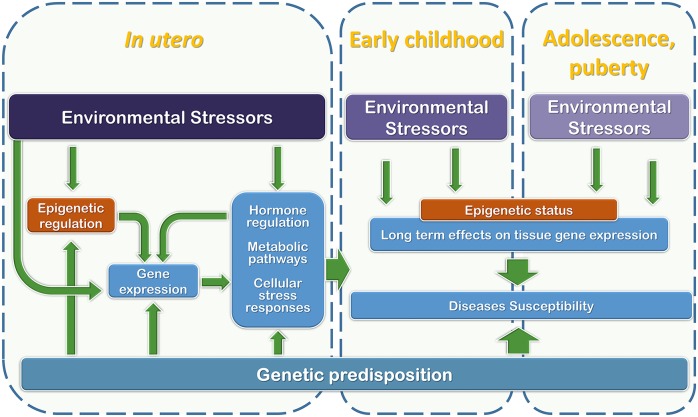
Graphical representation of the relationships among development phases, genetic predisposition, and environmental stressor factors that may interfere with regulatory mechanisms during the early stages of life (from *in utero* development to puberty).

As discussed above, fetal life is one of the crucial time windows during which future health takes shape through a dynamic interaction between the maternal/external environments and developing organs and tissues (Figure 2). In this context, the effort to characterize the fetal exposome is a priority for determining future health and disease predisposition.

The placenta, as a sort of gestational logbook, is a useful organ for defining the exposome, as well as a valid target organ for molecular biomarkers. In fact, the placenta plays a key role in the maintenance of an adequate intrauterine environment as well as in signal transmissions from the fetus to the mother and vice versa (101). Nutrition supplies, gas exchange, endocrine, and immune regulation are guaranteed by the placenta. At the same time, it has a pivotal role in minimizing the quantity of environmental contaminants, toxins, pathogens, and maternal stress hormones reaching the fetus (102). Placental development begins during the first few weeks after fertilization; from this moment, the success of fetal development is dependent on an appropriate placentation and on the remodeling of maternal circulation to ensure its perfusion (103). Moreover, environmental toxicants from maternal blood can reach the fetus only by passing through the placenta, which is known to be a selective barrier (104). Placental cells express detoxification enzymes and antioxidant molecules which are involved in fetal protection against toxicants and free oxygen radicals (105). On the other hand, those chemicals which do not pass the placental barrier may accumulate in placental tissue, thus modifying its functions and indirectly affecting the fetus. The placenta is usually discarded after birth and can be sampled in a non-invasive procedure. It has been previously defined as a “black-box” event recorder due to its highly informative potential for summarizing the *in-utero* experience (106). From this point of view, placental multi-omics investigation (e.g., epigenomics, transcriptomics, and proteomics) could be considered an essential step for simultaneously testing exposure, effects, and susceptibility biomarkers in a single biological matrix—i.e., a valid proxy for the internal fetal exposome.

For example, with respect to placental exposure biomarkers, studies have shown that placental levels of Cd correlate to the expression levels of the Metallothionein gene (107). Another study found that placental expression of the arsenic transporter AQP9 was positively associated with maternal urinary As levels during pregnancy (108). However, these studies consider placental gene expression for exposure to a single pollutant. Only a few studies have tried to investigate two (109) or more co-exposures and their potential effects on placental physiology or functions (110). Deyssenroth and colleagues proposed an exposure regression analysis to derive metal mixture indices associated with placental networks, which in turn are associated with small-for-gestational age (SGA) status. They found that, among 19 metals tested, As and Cd levels are associated with SGA, and the effects of these metals persist even after accounting for the presence of correlated co-pollutants (110).

With regards to biomarkers of possible effects, in a study conducted by Ahmed and colleagues, a reduction in levels of placental T cells and alterations of cord blood cytokine concentrations were observed in a Bangladeshi population associated with high maternal As exposure (111). Along the same line, Lambertini et al. (112) detected a placental-specific imprinted gene expression panel associated with both maternal psychosocial stress during pregnancy and birthweight. The same authors, in a previous work, also showed that alterations of placental imprinted gene expression were associated with suboptimal perinatal growth and responsive to exposure to PCBs and DDE (113). Finally, in a recent epigenome-wide study, Maccani et al. identified evidence of hypomethylation of the EMID2 gene in association with *in-utero* Hg exposure. This altered methylation status was also found to be linked to adverse neurobehavioral outcomes during infancy (114).

In addition to data from placental examination and the bio-monitoring of multiple pollutants in maternal and cord blood (internal exposome), maternal data collected through questionnaires (diet, physical activity, lifestyle, stress factors, socio-demographic characteristics, and use of medication during pregnancy) and geo-spatial data associated with environmental monitoring stations could be used to define the external fetal exposome (both general and specific) and its association with postnatal health outcomes.

In the context of a highly polluted site, cohort studies concerning the fetal exposome may be useful for describing the complexity of chronic multi-toxicant exposure, socioeconomic determinants, and maternal life-style habits and their combined effects on the derived population of children. In the case of heavily contaminated sites, social and physical environmental toxicants tend to cluster in the most socially disadvantaged populations (115); thus, a better understanding of these complex interdependencies may help to prevent health disparities. Socioeconomic status during childhood has been found to have greater influence on adult DNA methylation profiles than socioeconomic status during adult life (116). Moreover, dietary lifestyle and micronutrient supplementation may play a role in maintaining DNA stability (117). Nevertheless, the studies on the combined effects of lifestyles/socioeconomic determinants and environmental pollutants are poorly represented in the literature. Indeed, it is well-established that maternal stress increases blood cortisol levels. The placenta is able to reduce the amount of cortisol that can reach the fetus through the 11β-HSD2 enzyme (118). At the same time, exposure to Cd influences the expression and activity of placental 11β-HSD2 (119). Thus, the contemporary presence of stressful conditions during pregnancy and Cd exposure can irreversibly affect the hypothalamic-pituitary-adrenal axis via fetal exposure to cortisol. Another example of the joint effects of the social and physical environment includes the interaction between NO_2_ air exposure and elevated social stress on increasing risk for childhood asthma (120).

Finally, it is essential to produce consistent data to uncover the fetal developmental windows and molecular pathways most vulnerable to the negative influence of toxicants. Discovering specific biomarkers of prenatal exposure, which may be predictive for the child health outcomes, will increase our ability to develop early diagnostic and prophylactic/therapeutic tools to be applied in pregnant women residing in highly contaminated areas.

## Existing Birth Cohorts on Prenatal Exposome and Methodological Aspects

Highly contaminated sites often present serious environmental contamination scenarios, where pollutants can persist in the environment for decades even after pollution sources are removed. Moreover, in these areas, interaction between environmental pollutants and other health determinants, such as social stress, poverty, and limited access to medical services, may coexist (121). To date, the need to reduce environmental exposures has been widely highlighted, focusing on the close link between human health and the environment, as well as the possible large-scale economic return (122). Birth cohorts are an approach allowing the integration of the exposures as a whole, including those related to socioeconomic status, with “omics” data from biological samples collected at birth and throughout life. One of the major advantages provided by birth cohort design is the assessment of the early life environment, studying in-depth several possible confounders and outcomes by means of questionnaires, follow-ups based on clinical evaluation, and biological samples collected at different time points.

The Project on *Human Early Life Exposome*—HELIX is the first attempt at developing a multistep statistical analysis approach based on different tools and methods, also integrating “omics” into the exposome. To do this, HELIX pooled six existing longitudinal population-based birth cohort studies in Europe, measuring the external exposome (individual and outdoor exposures), integrating the external and internal exposome (integrating molecular exposure signatures), and evaluating the impact of the early-life exposome on child health (also including the effect of multiple exposures) (32). HELIX measured over 200 single environmental exposures of concern for child health, allowing a detailed analysis of the structure of the early life exposome, including its correlations, patterns, and variability (123). On the same emerging line, the Project *Health and Environment-wide Associations based on Large Population Surveys*—HEALS was aimed at identifying the complex links among genes, environment, and many human diseases by means of a large collaborative action among existing cohorts in Europe (124).

Recently, Sarigiannis and Karakitsios, in the context of COST Action IS1408 on “Industrially Contaminated Sites and Health Network,” developed a model for the characterization of the exposome in children living close to a very large landfill area (125). With a design especially developed for a highly contaminated area, this project is an attempt to use the exposome paradigm to better understand the relationships that exist among the co-determinants of exposures and its effects on the health of exposed individuals and their progeny.

The exposure of pregnant women to environmental contaminants present in highly contaminated areas can severely impact the wellbeing of future generations. The Italian *International Centre of Advanced Study in Environment, Ecosystem and Human Health* (CISAS) project is aimed at understanding the chemical-physical and biological processes that regulate the distribution of contaminants in various environmental matrices, as well as their transfer to the ecosystem and the human body and consequent health *sequelae* (126). In the context of the CISAS project, the “*Neonatal Environment and Health Outcomes*” (NEHO) birth cohort has been established by enrolling pregnant women residing in these contaminated sites and in surrounding areas. The CISAS project evaluates pollutants in all the environmental matrices (air, soil, sediment, inland waters, and sea) as well as the food chain (fish, meat, eggs, milk, and dairy products, sampled from local producers of each evaluated area) within three heavily contaminated sites in southern Italy: two widely industrialized coastal sites characterized by petrochemical complexes and power plants and one disused industrial area. Environmental data will be linked to georeferenced maternal residences, also taking into account possible daily commuting to work. The NEHO questionnaires collect information on maternal pre-pregnancy and gestational health status, lifestyle, and socio-demographic characteristics, along with smoking habits and possible chemical exposures. The protocol includes the collection of maternal and cord blood, along with placental tissue at delivery (126). In the context of the NEHO cohort, measurements of the levels of toxicants will be taken from maternal and cord blood as well as the placenta. Because the placenta has an active role in fetal development, and the impairment of placental formation, differentiation, and/or function may affect fetal development, in the context of the NEHO cohort we will investigate the relationship between exposure to environmental toxic compounds (both HMs and POPs) and shifts in gene expression by means of a whole transcriptome analysis. Finally, follow up of the offspring will be conducted to uncover the possible consequences of specific toxicants. The follow-up on children will allow the evaluation of the possible relationship between the fetal exposome and long-term health outcomes. To this aim, after delivery, information is collected on newborns regarding use of medicine, characteristics of home environments, breastfeeding and nutritional outcomes (including growth), respiratory disease, metabolic disorders, neurocognitive development, infections and injuries, and hospitalizations (also collecting hospital discharge records). The main objectives of the NEHO cohort are: (1) to understand processes and mechanisms for the transfer of HMs and POPs from the environment and the ecosystem to the developing fetus, (2) discover specific placental biomarkers informative of fetal exposure, and (3) identify possible primary intervention strategies aimed at reducing fetal exposure. The implementation of these milestones could have an impact on the early detection of negative outcomes during childhood based on placental omics, as well as on our ability to prioritize intervention strategies.

## Conclusions

The fourth session of the United Nations Environment Assembly of the UN Environment Programme, Nairobi 2019, recommended increasing efforts to overcome common health-related challenges as well as addressing the role of pollution as a cause of disease (127). Accordingly, the health of pregnant women in heavily polluted areas is an absolute priority for public health strategies.

To this end, in our opinion, the use of birth cohorts in heavily polluted areas represents a great opportunity for a better comprehension of the mechanisms underlying the relationship between environment and human health, adopting the *in-utero* developmental phase as a useful time window for identifying the origin of health and disease in childhood and adult life. In this context, the human placenta represents a useful matrix for exploring fetal exposure to environmental contaminants and possible predisposition to adverse health effects later in life.

## Author Contributions

SR, GD, FB, SS, and FC made substantial contributions to the conceptualization and design of the study. SR, GD, FB, and FC are involved in study monitoring. All authors drafted and critically revised the manuscript for its content, and gave final approval of the version to be published.

## Conflict of Interest

The authors declare that the research was conducted in the absence of any commercial or financial relationships that could be construed as a potential conflict of interest.
